# Cinematic rendering in rheumatic diseases—Photorealistic depiction of pathologies improves disease understanding for patients

**DOI:** 10.3389/fmed.2022.946106

**Published:** 2022-08-03

**Authors:** Milena L. Pachowsky, Harriet Morf, David Simon, Verena Schönau, Larissa Valor-Mendez, Johannes Knitza, Filippo Fagni, Klaus Engel, Michael Uder, Axel Hueber, Christian Schmidkonz, Georg Schett, Arnd Kleyer

**Affiliations:** ^1^Department of Internal Medicine 3 - Rheumatology and Immunology, Friedrich-Alexander Universität Erlangen-Nürnberg and Universitätsklinikum Erlangen, Erlangen, Germany; ^2^Deutsches Zentrum Immuntherapie, Friedrich-Alexander Universität Erlangen-Nürnberg and Universitätsklinikum Erlangen, Erlangen, Germany; ^3^Siemens Healthineers, Erlangen, Germany; ^4^Institute of Radiology, Friedrich-Alexander Universität Erlangen-Nürnberg and Universitätsklinikum Erlangen, Erlangen, Germany; ^5^Division of Rheumatology, Klinikum Nürnberg, Paracelsus Medical University, Nürnberg, Germany; ^6^Department of Nuclear Medicine, Friedrich-Alexander Universität Erlangen-Nürnberg and Universitätsklinikum Erlangen, Erlangen, Germany; ^7^Department of Industrial Engineering and Health, Technical University of Applied Sciences Amberg-Weiden, Weiden, Germany

**Keywords:** cinematic rendering (CR), rheumatic and musculoskeletal diseases (RMDs), imaging, rheumatoid arthritis (RA), psoriatic arthritis (PsA), axial spondyloarthritis (axSpA), giant cell arteriitis (GCA), photorealistic

## Abstract

**Background:**

Patient education is crucial for successful chronic disease management. Current education material for rheumatic patients however rarely includes images of disease pathologies, limiting patients’ disease understanding. Cinematic rendering (CR) is a new tool that allows segmentation of standard medical images (DICOMs) into pictures that illustrate disease pathologies in a photorealistic way. Thus CR has the potential to simplify and improve the explanation of disease pathologies, disease activity and disease consequences and could therefore be a valuable tool to effectively educate and inform patients about their rheumatic and musculoskeletal disease (RMD).

**Objectives:**

To examine the feasibility of creating photorealistic images using CR from RMD patients depicting typical rheumatic disease pathologies and, in a second step to investigate the patient-perceived educational potential of these photorealistic images in clinical routine.

**Methods:**

We selected conventional, high-resolution (HR) and positron emission tomography (PET) computed tomography (CT) images of patients with rheumatoid arthritis (RA), psoriatic arthritis (PsA), axial spondyloarthritis (axSpA), and giant cell arteritis (GCA) that showed typical respective disease pathologies. These images were segmented using CR technique. In a prospective study, physicians used CR-enhanced and conventional original images to explain the depicted pathognomonic pathologies to patients with the respective rheumatic disease. Patients were then asked to complete a questionnaire evaluating the perceived usefulness of being presented with CR-enhanced images to better understand their underlying disease.

**Results:**

CR images were successfully generated from above mentioned CT methods. Pathologies such as bone erosions, bony spurs, bone loss, ankylosis, and PET-based inflammation could be visualized in photorealistic detail. A total of 79 patients (61% females) with rheumatic diseases (RA 29%, PsA 29%, axSpA 24%, GCA 18%) were interviewed and answered the quantitative questionnaire. Mean age was 55.4 ± 12.6 years. Irrespective of disease, all patients agreed or highly agreed that CR-based images help to improve disease understanding, should be shown at disease onset, provide a rationale to regularly take medication and would like to have access to their own CR-enhanced images.

**Conclusion:**

Conventional disease images can successfully be turned into photorealistic disease depictions using CR. Patients perceived CR images as a valuable addition to current patient education, enabling personalized disease education and potentially increased medication adherence.

## Introduction

Adherence to treatment and overall compliance is an important component for successful management of patients with rheumatic diseases (1). Educated and informed patients have a higher disease awareness, which results in a higher probability of good treatment outcomes. EULAR recommendations call for shared decision making as an integral part of treatment within coordinated care for patients with rheumatoid arthritis (RA). Education, information and training should be provided to patients continuously during all stages of the course of their rheumatic disease (2). This strategy is not limited to RA but accounts for other rheumatic diseases (RMDs) as well (3).

At diagnosis, treating physicians need to explain the patients about rather complex disease processes and the benefit of treatment in a usually short time. This information has to be transported in an understandable way to the patient as a lay person and address the fears in regard to disease and therapy. Information has to be delivered in an empathetic but also convincing manner. With growing shortage of specialists and time constraints, concise, and self-explanatory tools supporting the patient-physician communication may become increasingly important (4–7). Recommendations to improve patient education especially at crucial time points, such as diagnosis or initiation of therapy, have been formulated by a EULAR taskforce (3). Furthermore, educational programs have shown to successfully improve wellbeing and the patients’ expertise about their own disease (8).

High-quality disease images may constitute an important tool in patient education. In our previous work we investigated the acceptance and usability of 3D-prints of joints, showing typical changes related to RA, for patients (9). Utilizing segmented images from high-resolution peripheral quantitative computed tomography (HR—pQCT) scanner from peripheral joints of RA patients and controls we were able to create 3D printed models of the joints that depicted the characteristic bone erosions in great detail. When demonstrating these models to RA patients they were deeply affected and concerned and stated to rethink their attitude to medication intake. The vast majority of patients (88%) would have liked to see these kinds of images at the beginning of the treatment during the first consultation. This technique was further developed for other RMDs: 3D-printing models of the spine and pelvis from axSpA patients were created and not only used for patient education but also to teach medical students (10).

Cinematic Rendering (CR) is a new way to present medical images. CR enables a photorealistic illustration of pathologies in almost any procedure, which is based on image layers. It allows a plastic and self-explanatory visualization of anatomical structures and pathologies compared to standard segmentation programs (11, 12). Besides clinical applications CR has successfully been implemented as a teaching tool for anatomy for medical students (13). The majority of the students (95%) experienced cinematic rendering as helpful to better understand anatomy. However, in the context of patient education CR-based visualization of disease has not been used.

We therefore questioned whether (i) it is possible to create CR images from patient data of rheumatic diseases based in conventional-, high-resolution- positron emission tomography (PET) and computed tomography (CT) and (ii) whether CR images can be used as an educational tool in the physician- patient interaction.

## Materials and methods

### Image acquisition and cinematic rendering

Conventional CT scans of the spine and pelvis of patients with axial spondylarthritis (axSpA), high-resolution peripheral CT scans of patients with RA and psoriatic arthritis (PsA), and PET-CT scans of patients with large-vessel vasculitis (GCA) were performed during routine clinical practice. These images were acquired at the Rheumatology Department of the University Hospital Erlangen (HR-pQCT), the department of Nuclear Medicine (PET-CT) and the Bamberg Hospital (conventional CT). Conventional radiographs were provided from the Department of Radiology at the University Hospital Erlangen, serving as controls as they are normally shown to patients for disease explanation. CR was carried out in a cooperation with Siemens Healthineers. Data were postprocessed using a CR software. CR images were reconstructed using a prototype rendering software (Cinematic Rendering Version 1.5.3, syngo. *via* Frontier, Version VB30, Siemens Healthineers, Forchheim Germany).

### Patients

We performed a prospective, interventional study on consecutive patients with RA, PsA, axSpA, and GCA. Patients were recruited at the outpatient clinics of the rheumatology department. The group of patients was selected consecutively during a time period of one month (May 2021). Patients who were selected had their diagnosis confirmed prior to the study. No patients with initial diagnosis were included. After written informed consent and ethics approval (298_20B) patients were included into the study. Patients were systematically guided through the images by experienced doctors (MP, HM, JK, LVM, VS). A conventional image was used to explain the main pathology of the respective disease, followed by exemplary CR- images showing the respective pathology in photorealistic detail. This approach was carried out for RA, PsA, and axSpA but not for GCA, where only CR images were used as GCA cannot be visualized by conventional radiography.

### Questionnaire

A questionnaire was developed to evaluate the possible benefits of CR in patient education. This comprised 7 items, each of which had to be answered for agreement on a scale of 1–5. The following scaling was used: 1 = “do not agree at all” to 5 = “agree to a high degree.” The following seven questions were marked independently by the patients in the appropriate place for the selected answer: (1) Did you understand your disease in the provided Cinematic Rendering images? (2) Did you understand your disease better through the presentation using Cinematic Rendering images than with a normal X-ray image? (3) Did you understand your symptoms better after seeing the presentation of Cinematic Rendering images? (4) Should Cinematic Rendering images be shown at the onset of the disease to enable patients to visualize the potential end stage of the disease? (5) Would the Cinematic Rendering images have helped you to understand why you should take the medication at the beginning of the disease? (6) Do you think it would be reasonable to use this type of Cinematic Rendering to improve patients’ understanding of their disease? (7) Would you like it to view, if it were technically possible, your own images in the Cinematic Rendering technique?

### Statistics

Demographic data and the results of the questionnaire were analyzed using descriptive statistical analysis. Statistical analysis using SPPS was carried out by comparing averages using *t*-test and ANOVA analysis.

## Results

### Feasibility of cinematic rendering-based depiction of pathologies in rheumatic diseases

**FIGURE 1:**
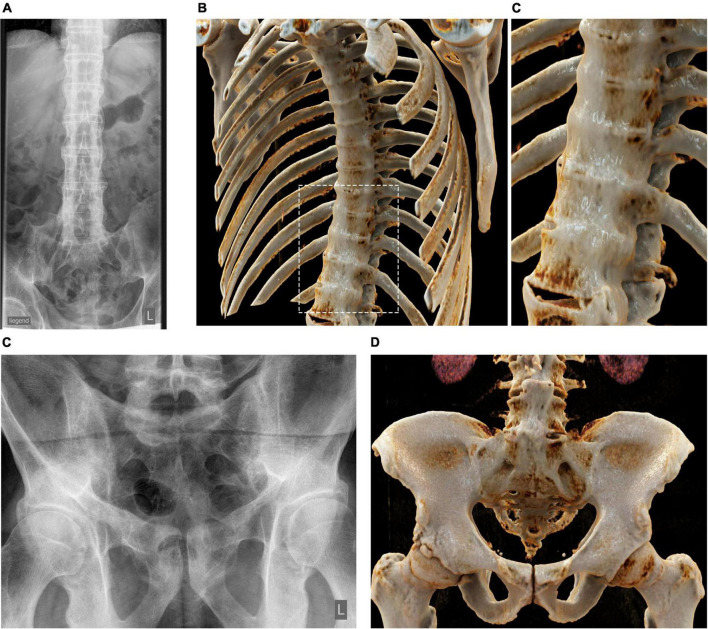
Cinematic rendering of the spine and ribs in axial spondyloarthritis (axSpA). **(A)** Conventional radiographs of the spine of a patient with axSpA showing syndesmophyte formation (bamboo spine). Cinematic rendering (CR) based on conventional CT images impressively illustrates the extent of the connected syndesmophytes wrapping around the thoracic spine and the ribs **(B)** and the lower thoracic spine where syndesmophyte formation is depicted in photorealistic way walling the spine [close-up **(C)**]. **(D)** Conventional radiographs of the pelvis of a patient with axSpA (left) showing sacroiliac ankylosis. Corresponding CR image showing the range of typical pathologies such as ankylosis or ubiquitous osteoproliferation along the iliac crest in photorealistic quality **(D)**.

**FIGURE 2:**
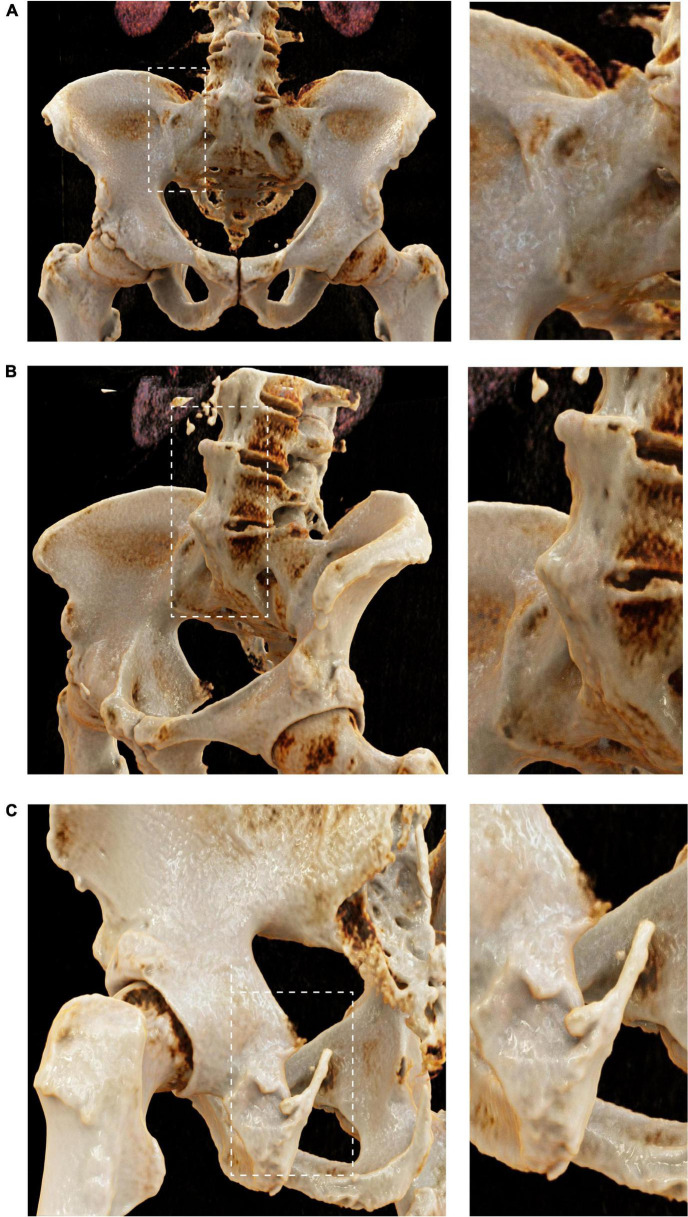
Cinematic rendering of the pelvis and sacrum in axial spondyloarthritis. Cinematic rendering based on conventional CT images impressively showing sacroiliac ankylosis [**(A)** overview and close-up], snydesmophyte formation at the ventral lumbar spine [**(B)** overview and close up] and enthesiophyte formation at the ischial tuberosity [**(C)** overview and close up]. CR close ups remarkably demonstrate the typical osteoproliferative pathologies, self-explaining the symptoms and pain these patients are suffering.

**FIGURE 3:**
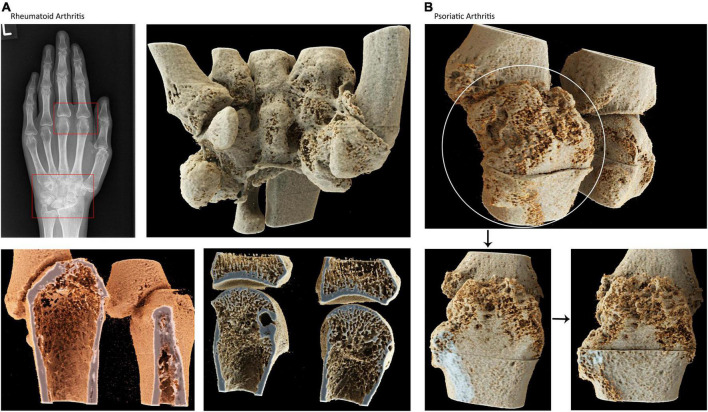
Cinematic rendering of the hand joints in rheumatoid and psoriatic arthritis. **(A)** Conventional radiographs of the hand of a patient with rheumatoid arthritis showing carpal and metacarophalangeal pathologies. Cinematic rendering based on high-resolution CT images showing **(B)** carpal bones with deformities and erosions (upper row) peripheral, trabecular demineralization and erosive changes in RA. This technique enables photorealistic depiction of the inner life of the human bone **(B)** cinematic rendering based on high-resolution CT images showing bone erosion and new bone formation in psoriatic arthritis self-explaining the difference between RA and PsA.

**FIGURE 4:**
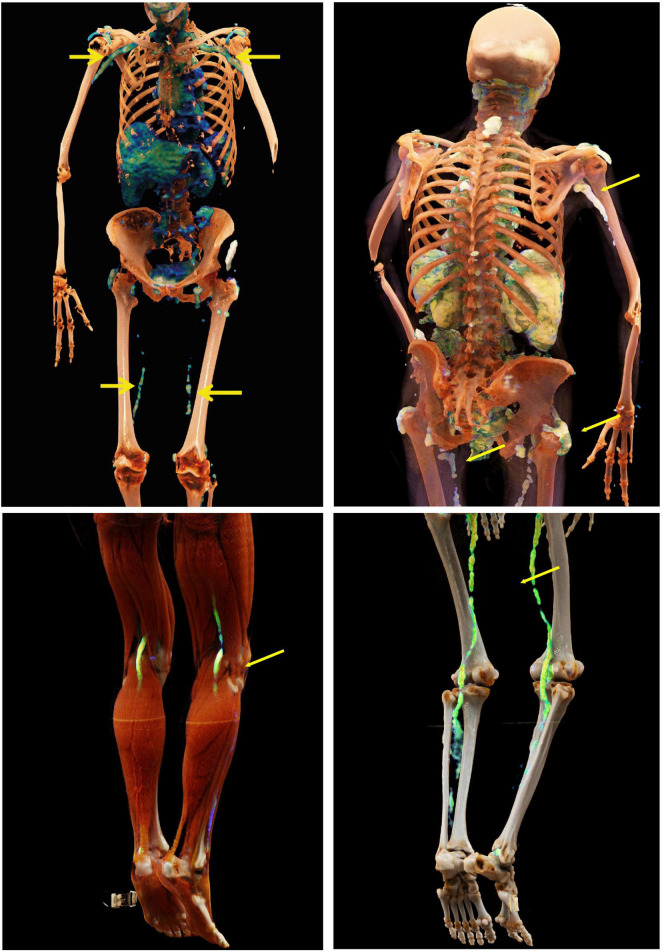
Cinematic rendering of the large vessels in giant cell arteritis. Cinematic rendering based on fusion images from PET and CT showing inflamed vessels (yellow arrows) in giant cell arteritis. Furthermore, inflammation of subacromial bursae is seen, which is associated with polymyalgia rheumatica. By applying CR on fusion images such as PET-CT scans, patients are easily able to understand the origin of inflammatory processes which are happening on vessel level in their body and thus understand why treatment is necessary.

We first created panels of CR-based images of the spine and ribs (from axSpA patients) (Figure 1), the pelvis and sacrum (axSpA) (Figure 2), the peripheral joints (RA and PsA) (Figure 3) and large vessels (GCA) (Figure 4). These panels allowed the visualization of syndesmophytes with bridging of the vertebral bodies, ankylosis of the sacroiliac joints, formation of bony spurs along enthesial sites, peripheral bone erosion and joint destruction as well as large vessel inflammation and stenosis in photorealistic quality. Patients with RA, PsA, axSpA, and GCA, described below, were exposed to these respective disease panels as well as with corresponding conventional radiographs of the spine, pelvis and hand joints.

### Patient characteristics

In total, 90 patients were approached and 79 patients agreed to participate in the study. Demographics of the participating patients are depicted in Table 1. *N* = 23 had RA, *N* = 19 axSpA, *N* = 23 PsA and *N* = 14 GCA. The mean age was 55.4 ± 12.6 years, 48 (60.8%) were female. The average disease duration was 8.81 ± 3.02 years. 79% had received immune modulatory therapy with bDMARDs, 16% with csDMARDs.

**TABLE 1:** Demographic data, disease duration and treatment.

Disease	*N*	Age (years ± *SD*)	Female (%)	Disease duration (years ± *SD*)	b/ts DMARD (%)	csDMARD (%)
Rheumatoid arthritis (RA)	23	56.33 ± 11.03	65.22%	11.43 ± 11.13	70	30
Psoriatic arthritis (PsA)	23	50.79 ± 14.77	52.17%	7.74 ± 10.88	83	17
Giant cell arteritis (GCA)	14	66.4 ± 8.6	85.71%	5.07 ± 4.60	50	14
Axial spondyloarthritis (axSpA)	19	52.11 ± 11.94	47.37%	11.11 ± 9.60	100	16
Total	79	55.4 ± 12.6	60.76%	8.81 ± 3.02	79	16

Shows demographic data, disease duration, and treatment data of the cohort. RA and PsA were the largest groups that participated, however patients with GCA were older in average with lowest disease duration. Of note 100% of axSpA patients received bDMARD or ts DMARD therapy.

### Reception of cinematic rendering images by patients

The overall reaction to the images was very positive (Table 2). Questions 1–7 were all answered very high with scores between 4.0 and 4.75 on a scale from 1 to 5. Question 1 aimed to analyze whether patients understood which alterations can be caused by their specific disease. On average in the whole group of all diseases, responses were high with a score of 4.39 (range 4.21–4.57). The answers to question 2 confirmed our hypothesis that patients understood their disease better using CR-based images than with conventional radiographs. Agreement was 4.43 (range 4.22–4.70). Question 3 was focused on whether the images helped to understand symptoms better. Agreements to this question were generally lower (as compared to questions 1 and 2) (4.24; range 4.00–4.48). Very strong approval was noted with question 4, which asked whether CR-based images should be shown at the onset of the disease to enable patients to visualize the potential end stage of the disease (4.40; range 4.26–4.50). Also, when patients were asked whether CR-based images help to understand why one should take medication from the beginning of the disease (question 5), the support was high in PsA patients (4.39) and GCA patients (4.25) but somewhat lower in RA (3.87) and axSpA (3.84). The question with the highest scores (4.68; range 4.53–4.78) in all groups (question 6) was about how reasonable it is to use CR-based images to improve patients’ understanding of their disease. Patients were also very interested to see their own images in by CR- technique (4.68; range 4.26–4.48).

**TABLE 2:** Results of the survey.

Disease	Question						
	1	2	3	4	5	6	7
Rheumatoid arthritis (RA)	4.35 ± 0.08	4.22 ± 1.13	4.00 ± 1.13	4.52 ± 0.99	3.87 ± 1.42	4.78 ± 0.52	4.26 ± 1.21
Psoriatic arthritis (PsA)	4.57 ± 0.95	4.70 ± 0.88	4.48 ± 0.99	4.26 ± 1.39	4.39 ± 0.94	4.65 ± 0.57	4.48 ± 1.08
Giant cell arteritis (GCA)	4.42 ± 0.67	4.42 ± 1.00	4.33 ± 0.98	4.50 ± 0.67	4.25 ± 0.97	4.75 ± 0.45	4.33 ± 1.37
Axial spondyloarthritis (axSpA)	4.21 ± 1.08	4.37 ± 1.01	4.16 ± 1.07	4.32 ± 1.16	3.84 ± 1.30	4.53 ± 0.70	4.32 ± 0.95
Total	4.39 ± 0.15	4.43 ± 0.20	4.24 ± 0.21	4.40 ± 0.13	4.09 ± 0.27	4.68 ± 0.11	4.35 ± 0.09

Shows the results of the survey. Average scores and standard deviation for each of the 7 questions per diagnosis group and for the whole group of patients are displayed. High scores where achieved for all questions in all diseases except of question 5. Here RA and axSpA patients showed slightly lower values to the question if CR images would have influenced them taking their medication right from the beginning of the disease.

## Discussion

Our study shows that it is possible to generate photorealistic images from typical pathologies observed in rheumatic diseases. These images highlight the structural damage and inflammatory burden of these diseases and demonstrate the importance of taking medication early in the course of disease. This was the first time, pathologic processes in SpA, PsA, and GCA were shown using Cinematic Rendering technique. Showing and explaining photorealistic images, as we conducted in this study, is an interactive way to improve patients’ understanding of disease and to strengthen the physician- patient interaction.

Our study demonstrates the high acceptance of patients for photorealistic images on rheumatic pathologies as an educational tool in the physician- patient communication with the potential to facilitate disease understanding. Patients with common rheumatic diseases understood their disease better by visualizing these images and agreed that the use of this kind of educational tool could improve patients’ perception of the disease. Especially the fact that patients understood their disease and the consequence of disease to joints, spine and vessels revealed the gap for patients’ needs and highlights clinical potential of CR. Other informational tools such as educational programs, books, leaflets, and web- based applications are accepted and beneficial but are usually used after the doctors’ visit. In contrast, the CR images can easily be shown during the appointment, providing the opportunity to directly explain the nature of disease, its consequences and the need for treatment (3).

Data showed that patients were able to easily recognize pathologies of their disease in CR images, perceive these alterations easier than in conventional radiographs and understand the origin of their clinical symptoms better, although we did not show them their own data. Deeper understanding of the disease achieved through the presentation of CR images can provide patients with a sense of self-efficacy, which in turn increases their trust in the treating physician. CR can thus play an important role in the future education of patients with rheumatic diseases. Furthermore, patients felt that imaging of late stage diseases when presented to them at disease onset could have been helpful to understand why to take medication at the beginning of the disease, especially among RA and GCA patients. Of note, a previous study showed that a structured patient education program for vasculitis patients can increase knowledge and improve quality of life but did not answer the question whether earlier education might have changed the start of drug therapy or patient adherence (14). Our results imply, that CR could close this gap.

We do not know from this study whether the use of CR-based images as an educational tool may improve the readiness of patients to take anti-rheumatic drugs and/or improve the adherence of patients on anti-rheumatic drugs. However, such effect might be assumed as earlier studies have shown that patients’ adherence improves when patients better understand their disease (15). Hence, in the future, structured implementation of CR-based images to assess its effect on patients‘ adherence will provide interesting results. Of note, patients seem to welcome to see their own disease depicted in a photorealistic way, which could add to individualized management and better adherence to treatment.

One of the limitations of the presented technique is the fact that it is not widely available yet. However, CR-based image processing only requires installation of a software and then can be used for many different imaging datasets. Furthermore, standard disease-specific CR-based images can be easily distributed and widely used for educational purposes. Demonstration of such images to patients is easy, fast and therefore feasible in daily clinical practice. It is also conceivable that in the future this data cannot only be offered *via* images but also through immersive technologies such as augmented reality, mixed reality (like *Hololens)* or virtual reality* (16, 17). This might even enhance the proven effect of those immersive applications in the context of teaching medical students or health care professionals improve their knowledge and disease understanding and thus empathize the patients’ symptoms and functional disabilities. Another limitation so far is the fact that in contrast to “pure” CT data, creation of CR-based images from fusion data, such as PET and CT, is still time consuming and requires additional computational power.

## Conclusion

Our study shows the feasibility to create CR-based images from imaging data of patients with rheumatic diseases and the high interest and acceptance of such data to educate patients with rheumatic diseases. Such CR-based images seem to be highly useful for educating patients about their diseases. Evidence based recommendations for education in patients with arthritis in general have been developed, addressing when, how, to whom, and by whom the education should be delivered and evaluated (2). Our data show that realistic three-dimensional imaging data like CR could complement and support such educational programs.

## Data availability statement

The datasets presented in this article are not readily available because of legal requirements. Requests to access the datasets should be directed to the corresponding author.

## Ethics statement

All patients gave informed consent—ethics approval 298_20 Ethics Committee FAU Erlangen-Nürnberg. The patients/participants provided their written informed consent to participate in this study.

## Author contributions

MP, AK, HM, JK, and GS wrote the main manuscript text. AK, AH, CS, and KE prepared the Figures 1–4. MP, HM, DS, VS, LV-M, JK, and FF included patients and conducted interview. All authors reviewed the manuscript.

## Abbreviations

axSpA: axial Spondyloarthritis
CR: Cinematic Rendering
CR: Conventional Radiography
CT: Computed Tomography
DICOM: Digital Imaging and Communications in Medicine
GCA: Giant Cell Arteriitis
HR-pQCT: High resolution peripheral computed tomography
MRI: Magnetic Resonance Imaging
PET-CT: Position Emissions Computed Tomography
RA: Rheumatoid Arthritis
RMD: Rheumatic Disease
PsA: Psoriatic Arthritis.
